# Trajectories of scholastic well-being: The effect of achievement emotions and instructional quality in the first year of secondary school (fifth grade)

**DOI:** 10.1007/s11218-022-09726-2

**Published:** 2022-08-30

**Authors:** Ramona Obermeier, Juliane Schlesier, Simon Meyer, Michaela Gläser-Zikuda

**Affiliations:** 1 Department for Educational Research, Linz School of Education, Johannes Kepler University Linz, Altenberger Str. 69, 4040 Linz, Austria; 2 Department of Educational Sciences, Primary Education, University of Greifswald, Steinbecker Str. 15, 17487 Greifswald, Germany; 3 Department of Education, Institute of Primary Education Research, University of Erlangen-Nuremberg, Regensburger Str. 160, 90478 Nuremberg, Germany; 4 Department of Education, School Education and Instructional Research, University of Erlangen-Nuremberg, Regensburger Str. 160, D-90478 Nuremberg, Germany

**Keywords:** Scholastic well-being, Achievement emotions, Instructional quality, Transition, Secondary school students

## Abstract

Due to their connections with positive educational outcomes, the maintenance of good health and scholastic well-being are highly relevant topics. However, to date, no studies have investigated developmental changes in scholastic well-being in the light of achievement emotions and instructional quality—although these predictors offer good approaches for intervention. A sample of *N* = 667 (age: *M* = 10.16; *SD* = 0.46; 81.7% female) students was questioned three times over one year, from the beginning of their fifth grade into sixth grade, using reliable questionnaires. The results of multilevel linear growth curve modelling calculations show that a decrease in scholastic well-being during the fifth grade was induced by changes in the predictors (achievement emotions and instructional quality). Since the highest effects are evident regarding achievement emotions, support programs should focus on facilitating these aspects in secondary school children.

## Introduction

Several studies affirm the positive effect of scholastic well-being on development, the maintenance of good health, and positive anticipation of critical life events (Choi, 2018; Hascher, 2008; Morinaj & Hascher, 2019; OECD, 2015, 2018). Scholastic well-being—as a facet of subjective well-being—is strongly associated with intrinsic motivation and thus with positive cognitive outcomes, such as higher academic achievement (e.g., Kleinkorres et al., 2020). Furthermore, studies have demonstrated an inseparable link between cognitive and affective aspects of learning that are considered to promote scholastic well-being as an independent educational goal (Hascher et al., 2018; OECD, 2018). In order to understand the affective and socio-emotional development of children, the scholastic setting offers a context of high potential (Morinaj & Hascher, 2019). Until now, longitudinal studies of trajectories of scholastic well-being have been rather neglected, especially with respect to shorter periods of time (e.g., one school year). The current study aims to provide insights into the development of scholastic well-being during the first year in secondary school (fifth grade), based on several factors that influence individual and scholastic levels. The researchers investigated the structure of hierarchically nested data by using multilevel growth curve models.

## Scholastic well-being and achievement emotions

### Scholastic well-being

Subjective well-being—implying personality growth and personal development—is a prerequisite for several factors in life, including health, social acceptance, adaption to requirements in several areas of life, and lifelong learning (Hascher et al., 2018; Morinaj & Hascher, 2019). Therefore, promoting well-being in various settings is a main goal of many social systems. Early concepts of well-being define a two-dimensional structure consisting of cognitive self-evaluations of emotional experiences in different settings (Diener et al., 2018). Following the assumption of Diener (1984), well-being can be described as affect-balance and thus as a continuum of positive and negative effects, which in turn, influence emotions and cognition (Diener et al., 2018).

Scholastic well-being, as a context-specific facet of subjective well-being defined above, concerns qualitative and quantitative cognitive evaluations of experiences in the school context, and is considered to be high when positive aspects outweigh negative perceptions (Hascher, 2008; Morinaj & Hascher, 2019; Schwab et al., 2022). The common definition of school well-being (Hascher, 2004b) includes cognitive, emotional, social, and physical components that are directly related to perceptions of the school environment or students' experiences of themselves therein. Student well-being at school is influenced by a variety of determinants and their complex interplay. The derivation of influencing factors is based on a fusion of individual-centered and environment-centered theories, and focuses on the fit of individual desires and needs with school requirements and conditions (Hascher, 2003, 2004b). Determinants of scholastic well-being that are considered to be universal are gender, educational track, and socio-economic and migration background (Hascher & Hagenauer, 2020; Herke et al., 2019; Morinaj & Hascher, 2019; OECD, 2018). Further determinants, such as social relationships, achievement emotions, self-concept and academic achievement, can be characterized as more processual and are mutually influenced by scholastic well-being (Choi, 2018; Putwain et al., 2020).

Emotions, in particular, are considered to be a fundamental element of both subjective well-being and scholastic well-being (e.g., Hascher, 2003). Today, scholastic well-being is a highly relevant issue in educational research and a self-contained goal of school administrations (Bonell et al., 2014; Borgonovi & Pál, 2016; Choi, 2018; Morinaj & Hascher, 2019; OECD, 2015, 2018).

### Achievement emotions

In general, emotions may be described as multi-component phenomena comprising coordinated psychological processes that include: physiological components (e.g., increased cardiovascular activity); cognitive components (e.g., worries); expressive components (e.g., an angry facial expression); and motivational components (e.g., an impulse to escape from a negative situation;Frenzel et al., 2015; Scherer, 2009). The component that distinguishes them from pure cognitive thoughts is the *core affect* (Russell, 2003; Schutz et al., 2010). Emotions that are experienced in learning and performance contexts are defined as *achievement emotions* (Frenzel & Goetz, 2018; Reisenzein, 2018; Schlesier, 2020). In most recent research in the field of educational psychology, classifications of achievement emotions according to valence (positive vs. negative), activation (activating vs. deactivating), and object focus (retrospective, current, prospective) have prevailed (Pekrun & Stephens, 2010). Furthermore, the differentiation between achievement-related activities (*activity emotions*) or achievement-related outcomes (*outcome emotions*) is well established (Pekrun, 2006, 2017). Activity emotions are experienced during learning (e.g., enjoyment, boredom) and outcome emotions are experienced in connection with success or failure, e.g., hope, pride, anxiety or shame (Pekrun, 2006, 2017).

The distinction between activity and outcome achievement emotions stems primarily from Perkun’s control-value theory (CVT) that has guided research on learning and instruction in the last two decades (Pekrun, 2006; Pekrun & Stephens, 2010). CVT assumes that it is not the learning or performance situation itself that determines the quality of the emotion experienced, but rather the interpretation of this situation (so-called appraisals;Pekrun & Stephens, 2010). Perceived subjective appraisals are therefore decisive for the emotion experienced in a triggering situation (e.g., “Do I control this situation self-determined, internally or am I externally controlled?”); and the subjectively attributed importance of the situation (value) (e.g., personal significance of the learning outcome; Frenzel & Goetz, 2018; Pekrun & Stephens, 2010). According to CVT, the environment is also decisive for the development of achievement emotions, for example, with regard to feedback, goal structure, and autonomy support (Pekrun & Stephens, 2010). Well-being is also frequently mentioned—but rarely assessed or examined—in relation to achievement emotions (with a few exceptions, e.g., Raccanello et al., 2020). Thus, there is a need for a study that encompasses achievement emotions, well-being and instructional quality.

### Conceptualization of scholastic well-being and achievement emotions

Although there is a link between emotions and well-being, and some early definitions and approaches equate the two constructs, some characteristics indicate distinct separation between them (overview in Hascher, 2008). Firstly, well-being is a broader category that includes an absence of physiological discomfort and social problems. Secondly, there are differences in the formation processes of well-being and emotions: the development of well-being is a slow process that is affected by the intensity and frequency of both positive and negative emotions experienced in any particular context (Hascher, 2003). By contrast, achievement emotions develop over a shorter period of time and are more perceptible than well-being in general (Ryan & Deci, 2001). Hence, Hascher (2004a, 2004b) presents a definition of scholastic well-being as a feeling based on one’s “own quality of experience, own expression and own physiological components” (Hascher, 2004a, p. 46).

Since students spend a significant amount of time participating in school and extra-curricular activities, particular focus should be placed on the school context when studying the affective development of children (Morinaj & Hascher, 2019). Aspects that are conducive to high scholastic well-being are related to one’s basic needs of autonomy, competence and social relatedness (Ryan & Deci, 2002). Scholastic well-being depends on both objective, contextual conditions that enable the fulfillment of basic needs, and the subjective perceptions of those conditions (Borgonovi & Pál, 2016; Morinaj & Hascher, 2019; Ryan & Deci, 2002). Circumstances that enable the fulfillment of a person’s needs are those which foster personality growth, life satisfaction, and well-being (Choi, 2018; Hascher, 2003; Hascher et al., 2018). Particularly during the first year in secondary school—which is accompanied by several changes in cognitive, social and organizational frameworks (e.g., higher academic requirements, new classmates, subject teachers)—students may suffer from a discrepancy between their basic needs and opportunities to fulfill them (Dawes et al., 2020; van Ophuysen, 2009). According to the theory of person–environment fit, this mismatch can be detrimental to scholastic well-being (Dawes et al., 2020).

## Current research findings on scholastic well-being, achievement emotions and instructional quality

Knowledge about the reciprocal relationships between affective and cognitive learning outcomes and multiple determinants of scholastic well-being is increasing, due to a significant amount of recent research on the topic (Borgonovi & Pál, 2016; Hascher & Hagenauer, 2020; Hascher et al., 2018; Morinaj & Hascher, 2019). Scholastic well-being is thought to be determined less by single aspects and more by the interplay of individual and contextual circumstances (Choi, 2018; Hascher, 2003). However, findings remain inconsistent, since they depend on the measurement of well-being and the highly subjective nature of the construct.

### Scholastic well-being and achievement emotions

Affective experiences in the scholastic context (such as positive emotions) have a significant impact on scholastic well-being (Choi, 2018; Hascher, 2008; Hascher et al., 2018; Morinaj & Hascher, 2019). Positive emotions (such as enjoyment) are conducive to well-being, which is an integral component of scholastic well-being; on the contrary—as might be expected—negative emotions have been shown to impair scholastic well-being (Hascher, 2008; Hascher & Hagenauer, 2011b; Obermeier, 2021). Previous research (e.g., Borgonovi & Pàl, 2016; Hascher, 2004a) on the impact of negative emotions on scholastic well-being addresses mainly the effects of anxiety or test anxiety. High levels of anxiety may lead to less favorable social, emotional and behavioral development, and lower attachment to school and school-related tasks (Borgonovi & Pàl, 2016). Findings on the impact of other negative achievement emotions, for example, hopelessness and boredom, are rare. However, there are some studies (although inconclusive) regarding the relationship between activity emotions and well-being of students (e.g., Camacho-Morles et al., 2021; Furlong et al., 2021; Raccanello et al., 2020) that analyze the relevance of boredom and other negative emotions—besides the impact of anxiety—for scholastic well-being.

### Scholastic well-being and instructional quality

Besides the determinants of individual scholastic well-being mentioned above, a large number of findings illustrate the powerful impact of instructional and social characteristics in the classroom. Previous studies reveal the beneficial effects of positive relationships with teachers and peers, high quality instruction, and greater possibilities for interaction with peers and learning materials. All these factors lead to a positive climate in the class, as well as increased well-being for students (e.g., Choi, 2018; Hascher, 2007; Hascher & Hagenauer, 2020; OECD, 2018).

Further key factors that are often recommended to induce scholastic well-being and positive achievement emotions, are a clear instructional structure and a student-centered approach (Choi, 2018; Gläser-Zikuda et al., 2005; Hagenauer et al., 2014; Kutsyuruba et al., 2015; Morinaj & Hascher, 2019; Obermeier, 2021; Obermeier et al., 2021). Self-regulated and active learning are conducive to fostering scholastic well-being, as well as feelings of deep understanding of the learning content. Thus, learning environments that provide opportunities for autonomous learning (without causing strain) and perceptions of positive relationships with classmates and teachers are to be encouraged (Choi, 2018; Hascher & Hagenauer, 2020). However, it is complicated to draw a coherent picture, since as may be expected, certain characteristics which are supposed to be objective are often perceived in a subjective and individual manner (Hascher, 2003, 2008).

### Development of scholastic well-being and achievement emotions

Prior findings on trajectories of scholastic well-being and achievement emotions show a relatively constant decrease as a child moves through the school system (Hascher & Hagenauer, 2011a, 2011b, 2020). Research in the German-speaking context implies that this negative trend starts in primary school (Wustmann et al., 2016) and continues throughout a student's school career (Eder, 2007; Hascher & Hagenauer, 2011a; Obermeier & Gläser-Zikuda, 2022). Nevertheless, there is a positive trend in scholastic well-being shortly after the transition to secondary school, where positive transition expectancies and positive emotions are well-known, especially for students entering middle or higher educational tracks (Hagenauer et al., 2014; van Ophuysen, 2008, 2009). In spite of the fact that initial levels of scholastic well-being are higher after transition to middle and academic tracks, there is also evidence of greater deterioration thereof among students in these groups (Knoppick et al., 2017).

Findings with regard to short-term changes in scholastic well-being are rather rare. Previous studies address scholastic well-being mainly in primary education (Wustmann et al., 2016), or in higher classes (sixth grade or higher) in secondary education (Hascher & Hagenauer, 2011a, 2011b, 2020). Research on trajectories of scholastic well-being—especially in the context of recent transition to middle and academic educational tracks—is scarce, as are studies that address changes within one school year.

## Research questions and hypotheses

Regarding prior findings, little is known about short-term changes in scholastic well-being, in particular about the period of time after the transition from primary to secondary school. Beyond that, it has not yet been investigated how potential changes in scholastic well-being may be induced by achievement emotions and instructional quality. Thus, this study aims to explore: (1) changes in scholastic well-being from the beginning of the fifth grade until the beginning of the sixth grade; (2) the influence of achievement emotions on scholastic well-being; and (3) the effects of perceived instructional quality. In order to reach these aims, three research questions arose and three hypotheses were tested:

RQ1: How does students' scholastic well-being change over the course of their first year in secondary school?

### H1

Based on prior studies by Herke et al. (2019), we may assume that children’s levels of scholastic well-being decrease during the first year of secondary school, after an initial positive trend.

RQ2: How is students’ scholastic well-being influenced by achievement emotions and their development?

### H2

Since previous studies confirm positive correlations of achievement emotions and well-being—especially for positive emotions such as enjoyment (Hagenauer et al., 2014; Hascher & Hagenauer, 2011a)—it can be expected that this decrease in scholastic well-being is substantially influenced by decreasing positive achievement emotions (Meyer & Schlesier, 2021).

RQ3: How does instructional quality influence the scholastic well-being of first-year secondary school students?

### H3

Based on existing findings (e.g., Choi, 2018; Hagenauer et al., 2014), it can be assumed that perceived decreasing teaching quality has a significant negative impact on the development of students’ scholastic well-being.

## Method

### Design and participants

The data were collected within the framework of a school development and evaluation project in secondary schools in southern Germany, from 2017 to 2019.^1^ The data presented here relate to mainly female students who had chosen either the medium or the academic track. Participation in the project was voluntary, anonymous and followed the guidelines of data protection. The students obtained informed consent from their parents. All respondents were informed about the objectives of the research project before the survey was administered. Ethics approval was not required, as per applicable institutional and national guidelines and regulations.

The study is based on a sample of *N* = 667 secondary school students who participated in three survey periods: October 2017 (t1), six weeks after they entered fifth grade (first year of secondary school in most German federal states); April/June 2018 (t2) in the second half of the fifth grade; and October 2018 (t3), about six weeks after the start of their second year in secondary school (the sixth school year in relation to the entire 9 to 13-year school career). Thestudents students were distributed among 53 classes across 19 catholic secondary schools, in the middle (57.2%) or academic track (42.8%). The average age of the students at the first measurement point was *M* = 10.19 (*SD* = 0.46). The sample included *n* = 544 girls (81.7%) of whom 57.4% (*n* = 382) were in single-sex schools. The percentage of students with a migration background^2^ was 24.8%, while the socio-economic status^3^ of 2.7% of them can be considered as low.

### Instruments

#### Scholastic well-being

Scholastic well-being was measured using a global score (Cronbach’s α: 0.89 ≤ α ≤ 0.91) calculated on five out of six dimensions of the Student Well-Being Questionnaire (SWBQ) developed by Hascher (2004b, 2007). Each criterion in the SWBQ comprises five to seven items. The positive dimensions of scholastic well-being include items on positive attitudes towards school (7 items; e.g., “I like to go to school.”); and positive academic self-concept (5 items; e.g., “I don't have problems mastering the tasks in school.”). The absence of negative dimensions of scholastic well-being include items on social problems in school (5 items; e.g., “I had no problems with my classmates during the last few weeks.”); worries in school (5 items; e.g., “During the last few weeks I didn't have to worry about handling the school reality.”); and physical complaints in school (6 items; e.g., “During the last few weeks I never had a headache in school.”). Prior to the calculation of a global value, the negative dimensions of scholastic well-being (absence of social problems, worries and physical complaints in school) were reverse coded. Since high values on all dimensions indicate a high state of well-being, a summative index was then computed.

#### Achievement emotions

Negative and positive achievement emotions were assessed using the mathematics-specific AEQ-M questionnaire (Pekrun et al., 2005) that was adapted to capture non-subject-specific achievement emotions in class: *anxiety*: 15 items (0.90 ≤ α ≤ 0.93), e.g., “I worry if this is all too difficult for me.”; *hopelessness*: 6 items (0.86 ≤ α ≤ 0.92), e.g., “During the test I feel hopeless.”; *boredom*: 6 items (0.89 ≤ α ≤ 0.82), e.g., “My homework bores me to death.”; *pride*: 6 items (0.84 ≤ α ≤ 0.88), e.g., “After a test I am proud of myself.”; and *enjoyment*: 10 items (0.84 ≤ α ≤ 0.87), e.g., “I enjoy my class.”. A five-point Likert-scale (1 = strongly disagree to 5 = strongly agree) was used to collect responses to all these items. The considerations that led to the use of a global instrument for the assessment of emotions are explained in the discussion.

#### Instructional quality

The study assessed instructional quality in class using an instrument developed by Lenske (2013): *structure*: 5 items (0.83 ≤ α ≤ 0.85), e.g., “I always know what to do in class.”; *climate*: 6 items (0.86 ≤ α ≤ 0.88), e.g., “The teacher is friendly to me.”; *classroom management*: 5 items (0.72 ≤ α ≤ 0.85), e.g., “I follow the rules in class.”; *activation*: 6 items (0.69 ≤ α ≤ 0.72), e.g., “I always work with concentration.”; and *balance*: 3 items (0.81 ≤ α ≤ 0.83), e.g., “I learn something in class.”.

### Statistical procedures

Prior to evaluating the trajectory of scholastic well-being and its influencing factors, missing data that partially exceeded 25% was deleted using a listwise approach. All analyzes were carried out using RStudio (version 4.0.4).

Firstly, to maintain an overview over the data, statistical correlations of each criterion with its various predictors were tested by means of Pearson’s coefficient. The function *reshape ()* was used to create a dataset in long format (with one row per measurement-point and participant) in order to transform the format of the data. Using a multilevel growth curve modelling approach (Field, 2012, 2018; Finch et al., 2014), several models were tested: First, an unconditional baseline model (Model 1) (without consideration of change over time) was fitted to evaluate the difference of the grand mean of the intercept from zero. Secondly, using the *lme()* function, a random intercept model (Model 2) was calculated. Next, time was added as a predictor (Model 3). Then, an unconditional random-intercept-random-slopes model (Model 4) was tested to investigate if intercepts and slopes of the students vary around the overall model.

In additional models, predictors were included stepwise: Model 5 represents a random intercept-random-slope model with achievement emotions as predictors; Model 6 includes aspects of instructional quality as predictors. The final model (Model 7) integrates both aspects as predictors of the development of scholastic well-being simultaneously in a random-intercept-random-slope model. Multilevel regressions were computed using the packages *nlme* (version 3.1–152). The random slope model was further specified into several conditional growth models with varying covariates. Lastly, the models were compared by means of ANOVA and AIC, BIC and log-likelihood methods were used to compromise the models. Hence, random effects were tested and the covariance structure of the data was investigated to reduce the Type I or Type II error rates. The first-order autoregressive covariance structure with the highest correlations at adjacent measurement points was assumed and checked (Field, 2018).

## Results

Correlation analyses show that there are high, significant correlations between scholastic well-being and the other variables. In particular, negative emotions and well-being are highly negatively correlated (see Table 1; hopelessness *r* = −0.78, anxiety *r* = −0.80, boredom *r* = -0.59). On the other hand, positive correlations are found between well-being and positive emotions (pride *r* = 0.49, enjoyment *r* = 0.56), as well as for well-being with all dimensions of instructional quality (*r* ranging from 0.46 to 0.66). Medium to high positive intercorrelations are found among negative emotions (e.g., anxiety and hopelessness, *r* = 0.82); among positive emotions (pride and enjoyment, *r* = 0.61); and among the instructional quality subscales (e.g., structure and balance; 0.55 ≤ *r* ≤ 0.77). Negative and positive emotions correlate respectively negatively and positively with the scales of instructional clarity.

**Table 1:** Means, standard deviations, and correlations with confidence intervals

Variable	*M*	*SD*	1	2	3	4	5	6	7	8	9	10
*Achievement emotions*												
1. Anxiety	2.21	0.64										
2. Hopelessness	1.85	0.68	.82**									
			[.80, .85]									
3. Boredom	1.94	0.70	.51**	.55**								
			[.46, .57]	[.49, .60]								
4. Pride	3.48	0.66	−.37**	−.48**	−.40**							
			[−.43, −.30]	[−.54, −.42]	[−.46, −.33]							
5. Enjoyment	3.27	0.48	−.44**	−.48**	−.64**	.61**						
			[−.50, −.37]	[−.54, −.42]	[−.68, −.59]	[.56, .65]						
*Instructional quality*												
6.Structure	3.73	0.60	−.48**	−.50**	−.54**	.45**	.56**					
			[−.53, −.42]	[−.55, −.44]	[−.59, −.48]	[.39, .51]	[.50, .61]					
7. Climate	3.88	0.63	−.44**	−.46**	−.56**	.42**	.52**	.77**				
			[−.50, −.38]	[−.52, −.40]	[−.61, −.51]	[.36, .48]	[.46, .57]	[.74, .80]				
8. Classroom management	3.46	0.57	−.42**	−.46**	−.47**	.40**	.49**	.69**	.75**			
			[−.48, −.35]	[−.51, −.39]	[−.53, −.41]	[.33, .46]	[.43, .54]	[.65, .73]	[.71, .78]			
9. Activation	3.40	0.45	−.33**	−.38**	−.44**	.52**	.57**	.63**	.58**	.55**		
			[−.40, −.26]	[−.44, −.31]	[−.50, −.38]	[.46, .57]	[.52, .62]	[.58, .67]	[.52, .62]	[.50, .61]		
10. Balance	3.82	0.68	−.45**	−.52**	−.64**	.54**	.69**	.69**	.66**	.60**	.63**	
			[−.51, −.38]	[−.57, −.46]	[−.68, −.59]	[.49, .60]	[.64, .72]	[.65, .73]	[.61, .70]	[.55, .64]	[.58, .68]	
11. Well-being	3.86	0.52	−.80**	−.78**	−.59**	.49**	.56**	.63**	.61**	.56**	.46**	.66**
			[−.82, −.77]	[−.81, −.75]	[−.64, −.54]	[.43, .55]	[.50, .61]	[.58, .67]	[.56, .66]	[.51, .61]	[.40, .52]	[.61, .70]

*M* and *SD* represent mean and standard deviation respectively. Values in square brackets indicate the 95% confidence interval for each correlation. The confidence interval is a plausible range of population correlations that could have caused the sample correlation (Cumming, 2014). * indicates *p* < .05. ** indicates *p* < .01

Overall, the growth curve models show that well-being decreases over time, which is particularly evident in both the random intercept model (Model 3), and the random-intercept-random-slope model (Model 4) in which only *time* is included as a predictor. The variable *time* is significant in both models 3 and 4, with a medium effect size (β = −0.12, *SE* = 0.01); no other predictors are included in either of those models. The inclusion of only emotions (Model 5) or instructional quality (Model 6) lead to a decrease in the effect size of the time variable, until it is no longer significant in the full model (Model 7).

The growth curve model analyses reveal that the full model with all predictors—as well as the random-intercept-random-slope structure (Model 7)—shows the best model fit indices (AIC = 684.87, BIC = 767.13, LogLik = −326.43, *F*(16) = 766.34, *p* < 0.001; see Table 2). This indicates that the development of scholastic well-being in the first year of secondary school cannot be explained by the variable of time, but exclusively by achievement emotions and instructional quality, and their development over time. Since Model 7 shows significantly better fit indices than all the other models (models 1–6), it can be deduced that the decrease in the students’ well-being in the first year of secondary school can be explained solely by changes in their own emotions (in particular, negative emotions), and the perceived instructional quality offered by the teachers.

**Table 2:** Growth curve models with several predictors of scholastic well-being

	Baseline model	Model 2	Model 3	Model 4	Model 5	Model 6	Model 7
Intercept	3.92^***^ (0.02)	3.92^***^(0.03)	4.16^***^ (0.04)	4.16^***^(0.04)	4.62^***^(0.10)	1.97^***^(0.10)	3.64^***^(0.12)
Time			−0.12^***^(0.01)	−0.12^***^(0.01)	−0.03^**^(0.01)	−0.03^**^ (0.01)	−0.02 (0.01)
Anxiety					−0.37^***^ (0.02)		−0.33^***^ (0.02)
Hopelessness					−0.12^***^ (0.02)		−0.10^***^ (0.02)
Boredom					−0.06^***^ (0.01)		−0.01 (0.01)
Pride					0.06^***^ (0.02)		0.04^**^(0.02)
Enjoyment					0.09^***^ (0.02)		0.02 (0.02)
Structure						0.12^***^(0.02)	0.06^**^(0.02)
Climate						0.15^***^ (0.02)	0.08^***^ (0.02)
Classroom management						0.09^***^ (0.02)	0.07^***^ (0.02)
Activation						−0.02 (0.03)	−0.05^*^ (0.02)
Balance						0.19^***^(0.02)	0.10^***^ (0.02)
AIC	2343.66	1920.36	1839.78	1807.20	873.42	1284.49	684.87
BIC	2353.95	1935.79	1860.34	1838.05	929.97	1341.05	767.13
Log Likelihood	−1169.83	−957.18	−915.89	−897.60	−425.71	−631.25	−326.43
N (obs.)	1263	1263	1263	1263	1263	1263	1263
N (groups: id)		421	421	421	421	421	421
		*F*(3) = 425.30^***^	*F*(4) = 82.59^***^	*F*(6) = 36.57^***^	*F*(6) = 981.51^***^	*F*(7) = 605.52^***^	*F*(16) = 766.34^***^

***p < .001; ^**^p < .01; ^*^p < .05

With the exception of time, boredom and enjoyment, all other predictors included in the full model (Model 7) show significant effects. The greatest effect is seen in the development of the emotion anxiety, which has a significant negative effect on the development of well-being (β = −0.33, *SE* = 0.02). Overall, negative emotions appear to have a greater impact on students’ well-being than positive emotions, as shown in Models 5 and 7. Among the negative emotions, boredom seems to have significantly lower impact on the development of well-being than the other negative emotions. Comparing the parameters and fit indices of models 5 and 6 reveals that emotions can explain the development of well-being better than the instructional quality subscales, as both AIC and BIC values show a better fit in the model that includes only emotions as predictors (Model 5). Regarding the impact of instructional quality, it can be seen that perceived climate and balance, in particular, are most crucial for the favorable development of students’ well-being.

## Discussion

### Theoretical significance

To sum up, this comprehensive study has revealed how children’s scholastic well-being develops over the course of the fifth grade, highlighting the importance of achievement emotions and instructional quality. As assumed and described in prior studies (Hascher & Hagenauer, 2011a; Herke et al., 2019), well-being decreases steadily during the course of the fifth grade, up to the beginning of the sixth grade. In this process, intra- and inter-individual differences can be detected. Without considering the effects of achievement emotions and instructional quality on the development of scholastic well-being, the negative trajectory postulated in H1 can be confirmed. Students’ scholastic well-being seems to decrease even within the first year in secondary school, which is in line with existing findings (e.g., Hascher & Hagenauer, 2020; Wustmann et al., 2016).

In contrast to prior longitudinal studies in children of secondary school age—that neglected processual characteristics of scholastic and individual achievement emotions (Hascher & Hagenauer, 2011a)—when including corresponding variables as predictors, this study found no evidence of a negative trajectory. Thus, research question 2 concerning the impact of achievement emotions on the development of scholastic well-being can be answered, and H2 can be confirmed. Referring to existing research on the decrease in positive achievement emotions and the increase in negative achievement emotions (e.g., Hagenauer et al., 2014; Meyer & Schlesier, 2021; van Ophuysen, 2009), the results contribute to the understanding that the trajectories of achievement emotions are significant in influencing the trajectory of scholastic well-being. In particular, the finding that it is primarily the drop in negative emotions that leads to a drop in academic well-being should be highlighted. Furthermore, the results underpin the effects of several aspects of instructional quality as being crucial for the development of scholastic well-being of students in the course of the first year in secondary school (H3).

Thus, the findings of the current study expand prior findings on the determination of scholastic well-being, by revealing the importance of individual negative achievement emotions of students, as well as the positive impact of instructional quality. Besides aspects of instructional quality in class, there are fundamental correlations between scholastic well-being and achievement emotions, which are in line with prior findings (Hagenauer et al., 2014; Hascher & Hagenauer, 2011a).

Even though both achievement emotions and aspects of instructional quality are already known to be crucial for the formation and development of scholastic well-being, the results of these analyses contribute to a deeper understanding of the trajectories of scholastic well-being. It is particularly relevant that this development in scholastic well-being cannot be explained over time, but is explained exclusively by the development in achievement emotions and perceived instructional quality, as can be seen in the current data. In this context, achievement emotions explain scholastic well-being more so than instructional quality does. Based on the growth curve model which shows the best fit (Model 7), we confirm that negative changes in scholastic well-being are significantly influenced by decreasing positive achievement emotions (Meyer & Schlesier, 2021) and instructional quality (e.g., Hascher, 2007).

### Limitations

The present study has some limitations that need to be addressed and discussed when interpreting the findings. Firstly, the surveyed group of students is a regional sample taken from catholic schools in southern Germany (e.g., Pirner et al., 2010), which restricts the ability to draw universally valid conclusions. Nevertheless, since the analysis of predictors of the development of scholastic well-being of students in private schools has so far been a desideratum, the present study offers considerable added value in this respect.

The imbalanced gender ratio—as many single-sex schools for girls were included in the sample—represents another limitation. Potential effects (or the absence thereof) should therefore be interpreted in the light of this limitation. Moreover, it should be noted that the study addresses students’ achievement emotions and scholastic well-being in general, which means that no inferences can be made about domain-specific differences between subjects (e.g., Frenzel et al., 2015). Although the subject-specificity of achievement emotions is well known (e.g., Goetz et al., 2006), we chose to exclude that aspect for several reasons: First, we wanted to get an overview of the emotional traits of the students. Second, we wanted to obtain the same level of abstraction as for the global measure of scholastic well-being. Third, the students had not yet had the opportunity to experience emotions—nor scholastic well-being—in the newly differentiated subjects, especially at the first time of measurement (six weeks after they entered fifth grade). Therefore, in future research, the development of scholastic well-being should be investigated taking different school subjects into account, to examine possible correlations and interactions between subject-specific learning conditions and the experience of scholastic well-being.

Lastly, it should be mentioned that the data was collected before the outbreak of the Corona virus pandemic, which is why the effects of the pandemic—for example, in terms of classroom structure and the use of media (Helm et al., 2021)—are not represented in the current study.

### Conclusion and implications

As empirical evidence on the importance of school well-being for academic achievement, educational participation, and personal development grows the need to promote the well-being of students at school is now accepted and pursued through various support programs (e.g., Bonell et al., 2014, Borgonovi & Pàl, 2016). In particular, programs to promote positive achievement emotions and to improve the quality of teaching seem to be an adequate way of achieving improved scholastic well-being.

Based on the findings of this study, children in the fifth and sixth grades should be the primary focus of support programs. Since especially anxiety and hopelessness have a significant influence on the development of scholastic well-being, the inhibition of these negative achievement emotions should be included in support programs. More broadly, training programs to support emotional competencies (e.g., Petermann et al., 2016) are required in order to enhance students’ repertoire of emotional regulation strategies. Social-emotional learning (SEL) programs can help children to enhance their emotional intelligence skills, such as the ability to identify positive and negative emotions (Reicher & Matischek-Jauk, 2018).

Besides student emotions, it should be noted that instructional quality is controlled primarily by the teacher, and is therefore easier to promote through ongoing teacher training. Instructional quality could be increased, for example, by creating a learning climate that is oriented towards the children in the class, and their particular needs. More autonomy, co-determination and self-regulation for children, and better classroom management, feedback and formative performance assessment by the teacher can be helpful starting points to improve instructional quality (e.g., Hattie & Timperley, 2007; Markus & Gläser-Zikuda, 2021; Schwab et al., 2022). Appropriate feedback from teachers is positively linked to achievement emotions and in turn, scholastic well-being. Thus, teachers should be aware of their role in the positive emotional experience of students and their influence on students’ well-being at school, and actively seek to promote these aspects in students (Schwab et al., 2022). In addition to the significant role of the teacher, the social climate also plays an important part in promoting positive achievement emotions and scholastic well-being of students. These goals can be fostered by actively promoting social cohesion and a sense of belonging in the classroom (e.g., Dawes et al., 2020). The provision of adequate feedback and improvement of social cohesion should be specifically addressed and supported in the training of teachers in terms of classroom management skills (e.g., Dawes et al., 2020; Schwab et al., 2022). These relations should be investigated in follow-up studies.

This study provides an important insight into the interrelationships between the stated variables in relation to achievement emotions, instructional quality and hence scholastic well-being. The results indicate that anxiety, hopelessness and pride have an effect on the development of scholastic well-being. Regarding various aspects of instructional quality, the highest effects are related to the climate and the students' assessment of having learned something. A more differentiated analysis would be beneficial in future research in order to derive more concrete implications for practice. For this purpose, achievement emotions should be analyzed in specific subjects, and the different dimensions of school well-being (e.g., Hascher, 2003, 2004b) should be examined in detail for their connections with emotions and teaching quality. Specific analyses could be connected to these achievement emotions as well as to the mentioned aspects of teaching quality.

## Footnotes

1 In most federal states in Germany, the school system comprises a four-year elementary school and subsequent transfer to various secondary schools at the beginning of grade five. Depending on their academic performance and the qualification they wish to achieve (vocational training or academic career), students can choose between a 5-year secondary school, a 6-year intermediate school, and a 9-year grammar school, also taking into account the recommendations of their primary school teachers. However, there are some differences between the federal states, which are not addressed in more detail in this study, since the data were collected in only one state.

2 A ‘migration background’ implies that at least one parent was born outside Germany.

3 Socio-economic status was measured using the ISCED-97 coding scale. Low socio-economic status was assumed if neither parent had attained the equivalent of at least the middle educational track or vocational school.
